# Unlocking the power of motor imagery: a comprehensive review on its application in alleviating foot pain

**DOI:** 10.1007/s13760-024-02492-2

**Published:** 2024-03-09

**Authors:** Roberto Tedeschi

**Affiliations:** Department of Biomedical and Neuromotor Sciences (DIBINEM), Alma Mater Studiorum, University of Bologna, Via Zamboni 33, 40126 Bologna, Italy

**Keywords:** Motor imagery, Foot pain, Cortical reorganization, Chronic pain, Rehabilitation

## Abstract

**Background:**

Motor imagery is a cognitive process that involves mentally simulating movements without physical execution. It has been studied in the context of foot pain to understand the role of motor cortical reorganization and its impact on motor imagery abilities. However, further research is needed to establish consistent evidence regarding the relationship between motor imagery and foot pain.

**Methods:**

This review analyzed five relevant articles that investigated motor imagery in the context of foot pain. The studies involved participants with various conditions, including leg amputation, chronic leg pain, complex regional pain syndrome, and Achilles tendinopathy. Different methodologies were employed, including motor cortical mapping, foot laterality recognition tasks, EEG recordings, and treatment interventions incorporating motor imagery.

**Results:**

The findings indicated that individuals with leg amputation exhibited functional reorganization in upper limb motor cortical maps, with a breakdown in the inhibitory relationship between foot and hand representations. Participants with chronic leg pain demonstrated slower and less accurate performance on foot laterality recognition tasks compared to healthy controls. Complex regional pain syndrome patients displayed distinct motor imagery strategies and responded differently to first-person and third-person perspectives. EEG studies revealed differences in brain activity during motor imagery tasks under pain-free and pain conditions. Treatment interventions incorporating motor imagery showed promising outcomes in improving functional outcomes and reducing pain levels.

**Conclusions:**

Motor imagery plays a significant role in foot pain conditions, although the evidence is still emerging. The findings suggest that motor imagery abilities may be affected by leg amputation, chronic pain, and complex regional pain syndrome. Further research is needed to establish standardized protocols for assessing motor imagery, identify specific patient populations that may benefit most from motor imagery interventions, and explore long-term effects. Integrating motor imagery into clinical practice has the potential to enhance rehabilitation approaches and improve outcomes in foot pain management.

## Introduction

Motor imagery, the cognitive process of mentally simulating movements without physical execution, has emerged as a promising intervention for pain management in various conditions. In particular, its application in alleviating foot pain has garnered increasing attention [1]. Foot pain can significantly impact an individual's mobility and quality of life, and traditional pain management approaches may not always provide satisfactory results [2–4]. Motor imagery offers a non-invasive and potentially effective alternative for addressing foot pain. The concept behind motor imagery is rooted in the understanding that the brain processes imagined movements in a similar manner to actual physical movements. By engaging in mental rehearsal of specific foot movements, individuals can activate and stimulate neural pathways associated with motor control, potentially influencing pain perception and promoting functional recovery [5, 6]. Motor imagery is believed to modulate cortical excitability, influence sensorimotor integration, and enhance body representation, leading to pain reduction and improved motor performance. This review aims to explore the current body of literature on motor imagery and its application in the context of foot pain [7–10]. By examining studies conducted in this field, we seek to investigate the effectiveness, underlying mechanisms, and potential benefits of motor imagery interventions for individuals experiencing foot pain. We will explore various aspects, including the selection of motor imagery tasks, the duration and frequency of practice sessions, and the integration of motor imagery with other therapeutic approaches [11–16]. Additionally, this review will delve into the neurophysiological mechanisms underlying the effects of motor imagery on foot pain. Understanding how motor imagery influences pain processing pathways and the neuroplastic changes it may induce can provide valuable insights into its therapeutic potential and guide the development of targeted interventions. By consolidating and analyzing the existing evidence, this review aims to provide clinicians and researchers with a comprehensive overview of the current state of knowledge regarding motor imagery in the context of foot pain [17]. The findings may contribute to the refinement and optimization of motor imagery interventions, ultimately leading to improved pain management strategies and enhanced functional outcomes for individuals suffering from foot pain. Furthermore, the utilization of motor imagery in foot pain management holds promise for individuals who may have limitations or contraindications for traditional physical interventions [18]. This non-invasive technique can be easily incorporated into rehabilitation programs, making it accessible to a wide range of individuals, including those with restricted mobility or sensitivity to touch. Moreover, motor imagery offers a patient-centered approach, empowering individuals to actively participate in their own pain management. By engaging in mental practice, individuals can gain a sense of control [19–21] over their pain experience and actively contribute to their own recovery [22]. This self-directed approach can enhance motivation, self-efficacy, and overall engagement in the rehabilitation process. The potential benefits of motor imagery extend beyond pain reduction [23]. Studies have suggested that it may also improve motor function, proprioception, and movement accuracy in individuals with foot pain. By rehearsing specific movements in their mind, individuals can enhance their neuromuscular coordination, leading to improved balance, gait, and functional performance. While motor imagery shows promise in the context of foot pain, it is important to acknowledge the need for further research and refinement of protocols. Standardization of motor imagery techniques, optimization of training parameters, and identification of patient-specific factors that influence its effectiveness are crucial areas of investigation. Additionally, exploring the long-term effects and durability of motor imagery interventions in foot pain management is essential for establishing its role in clinical practice.

## Methods

The present scoping review was conducted following the JBI methodology [24] for scoping reviews. The Preferred Reporting Items for Systematic reviews and Meta-Analyses extension for Scoping Reviews (PRISMA-ScR) [25] Checklist for reporting was used.

### Research team

To support robust and clinically relevant results, the research team included authors with expertise in evidence synthesis, quantitative and qualitative research methodology, sport and musculoskeletal rehabilitation.

### Review question

We formulated the following research question: "What is the current evidence regarding the effectiveness of motor imagery in the management of foot pain?".

This research question guided the identification, analysis, and synthesis of relevant studies, aiming to provide a comprehensive overview of the existing literature and gain insights into the potential advantages and limitations of motor imagery in the context of foot pain management.

### Eligibility criteria

Studies were eligible for inclusion if they met the following Population, Concept, and Context (PCC) criteria.

#### Population

The participants in the studies could include individuals of any age or gender who were experiencing foot pain due to various causes, such as musculoskeletal conditions, neuropathies, or other etiologies.

#### Concept

The concept of interest was the application of motor imagery techniques for managing foot pain. This could encompass various forms of motor imagery interventions, such as mental rehearsal of foot movements, visualization of pain reduction or healing, or other related techniques aimed at modulating pain perception or improving functional outcomes.

#### Context

The studies could be conducted in various settings, including clinical settings, rehabilitation centers, or research laboratories. The context of interest included both acute and chronic foot pain conditions, and studies could involve comparisons with control groups, different interventions, or pre- and post-intervention assessments.

By applying these Population, Concept, and Context (PCC) criteria, we aimed to ensure the inclusion of studies that specifically addressed the use of motor imagery techniques for managing foot pain, thus providing a focused and relevant synthesis of the available evidence.

### Exclusion criteria

Studies that did not meet the specific PCC criteria were excluded.

### Search strategy

An initial limited search of MEDLINE was performed through the PubMed interface to identify articles on the topic and then the index terms used to describe the articles were used to develop a comprehensive search strategy for MEDLINE. The search strategy, which included all identified keywords and index terms, was adapted for use in Cochrane Central, Scopus, PEDro. In addition, grey literature (e.g. Google Scholar, direct contacts with experts in the field) and reference lists of all relevant studies were also searched. Searches were conducted on 19 June 2023 with no date limitation.

### Study selection

After completing the search strategy, the search results were collected and imported into EndNote V.X9 (Clarivate Analytics). To ensure the accuracy of the dataset, duplicates were removed using the EndNote deduplicator, resulting in a file containing a unique set of records. This file was then made available to the reviewers for further processing. The selection process involved two levels of screening using the Rayyan QCRI online software12. At the first level, titled "title and abstract screening," two authors independently reviewed the articles based on their titles and abstracts. Any conflicts or discrepancies between the reviewers' decisions were resolved by a third author. The goal of this level was to assess the relevance of each article to the research question based on the provided information. The second level of screening, known as "full-text selection," also involved two authors independently reviewing the full texts of the selected articles. The purpose of this level was to assess the eligibility of each article based on its complete content. Again, any conflicts or disagreements between the reviewers were resolved through discussion and, if necessary, consultation with a third author. Throughout the selection process, detailed records were maintained, documenting the reasons for excluding articles that did not meet the inclusion criteria. This documentation followed the latest published version of the Preferred Reporting Items for Systematic Reviews and Meta-analyses (PRISMA 2020) flow diagram. The PRISMA flow diagram visually represents the screening process, indicating the number of articles identified (Tables 1, 2), screened, assessed for eligibility, and included in the final analysis. By adhering to these rigorous selection procedures and reporting guidelines, transparency and reliability were ensured in the article selection process, enabling a comprehensive and systematic approach to be taken in the scoping review.

**Table 1:** Main characteristics of included studies

N°	Author	Title	Year	Country	Study design	Source of evidence
1	Marconi B et al. [11]	Breakdown of inhibitory effects induced by foot motor imagery on hand motor area in lower-limb amputees	2007	Italy	Trial	Traditional
2	Coslett HB et al. [12]	Mental motor imagery and chronic pain: the foot laterality task	2010	USA	Trial	Traditional
3	King R et al. [13]	My foot? Motor imagery-evoked pain, alternative strategies and implications for laterality recognition tasks	2015	UK	Case Report	Traditional
4	Shamsi F et al. [14]	Recognizing Pain in Motor Imagery EEG Recordings Using Dynamic Functional Connectivity Graphs	2020	USA	Trial	Traditional
5	Shepherd M et al. [15]	The clinical application of pain neuroscience, graded motor imagery, and graded activity with complex regional pain syndrome-A case report	2020	USA	Case Report	Traditional
6	Rio EK et al. [16]	Implicit Motor Imagery of the Foot and Hand in People with Achilles Tendinopathy: A Left Right Judgement Study	2021	UK	Trial	Traditional

**Table 2:** Types of interventions

Study	Population	Method	Outcome
Trial	8 right leg amputees < br > 9 healthy subjects	Motor imagery tasks: imagined ankle dorsiflexion and plantarflexion Focal transcranial magnetic stimulation used to map hand/forearm muscle representations	In healthy subjects, motor imagery tasks inhibited map volume and contracted map area of hand muscles In amputees, imagined dorsiflexion and plantarflexion enhanced map area and volume of hand muscles Imagined dorsiflexion facilitated MEP amplitudes of extensor and inhibited flexor muscles of upper limb in both groups
Trial	40 subjects with leg pain (19 bilateral, 11 right, 10 left leg pain) < br > 42 subjects with chronic pain not involving the legs < br > 38 controls	All groups were slower and less accurate with stimuli requiring greater mental rotation of their foot	Subjects with leg pain were slower and less accurate than normal and pain control subjects in responding to drawings of a painful extremity Subjects with leg pain showed a greater decrement in performance for stimuli requiring larger amplitude mental rotations
Case Report	1 patient with CRPS Type II affecting the right foot	Laterality Recognition Task (LRT) using foot images to determine laterality (left or right)	Patient initially used a '3rd person' strategy (imagining someone else's feet) and performed well with 100% accuracy Patient was then asked to use a '1st person' strategy (imagining her own feet), which caused increased pain and slower response times Patient practiced LRTs at home and showed faster overall response times, but the same pattern remained (slower for right foot images) Biomechanical constraints analysis showed slower response times for awkward postures of the affected right foot with '1st person' strategy
Trial	Participants performing motor imagery tasks under pain-free and pain conditions	Functional connectivity-based feature extraction approach along with LSTM classifier employed for classification Four motor imagery classes considered: right hand, left hand, foot, and tongue	Average classification accuracy in the range of 77.86%-80.04% when considering all frequency bands First study demonstrating discrimination of motor imagery tasks under pain and pain-free conditions from EEG recordings
Case Report	A 57-year-old female with CRPS type 1	Functional Ankle Ability Measure (FAAM)	FAAM scores exceeded clinically important change sustained at two-year follow-up No functional deficits related to foot or ankle observed Minimal to no catastrophizing and fear avoidance behaviors
Trial	126 participants (40 unilateral AT cases, 22 bilateral AT cases, 61 controls)	Three independent studies with similar protocols were conducted by separate research groups. Each study-site evaluated left/right judgement performance for images of feet and hands using Recognise© software and compared performance between people with AT and healthy controls	No differences between AT cases and controls for hand image accuracy and reaction time No differences between AT cases and controls for foot image reaction time Conflicting findings for foot accuracy, based on four separate analyses No differences between the affected and unaffected sides in people with unilateral AT

*AT* Achilles tendinopathy, *CRPS* chronic regional pain syndrome, *EEG* electroencephalography, *FAAM* foot and ankle ability measure, *GMI* graded motor imagery, *LRT* laterality recognition task, *LSTM* long short-term memory, *MEP* motor evoked potential, PNE pain neuroscience education, *PT* physical therapy.

### Data extraction and data synthesis

Data extraction was conducted using a pre-designed data extraction form, specifically developed for this scoping review. The form was created based on the JBI (Joanna Briggs Institute) data extraction tool, tailored to capture key information from the selected articles. The extracted data included the following details: authors, country of publication, year of publication, study design, patient characteristics, pertinent findings or outcomes, type of intervention, related procedures, and any relevant additional information. Descriptive analyses were performed on the extracted data to summarize the characteristics of the included studies. The results were presented in a numerical format, using frequencies and percentages to report the studies identified and included in the scoping review. This approach allowed for a concise representation of the distribution and composition of the included studies. The description of the search decision process, including the number of articles identified, screened, assessed for eligibility, and ultimately included in the review, was systematically mapped. This mapping process provides transparency and clarity in documenting the selection process, allowing for a comprehensive understanding of the article selection flow. Importantly, the extracted data were summarized in tabular form, presenting the main characteristics of the included studies. These summary tables provide a structured overview of the key information extracted from each study, facilitating comparison and analysis of the findings across the included articles. Overall, the presentation of the extracted data in this scoping review primarily relies on concise and informative summary tables, providing a clear and organized representation of the main characteristics and results of the included studies.

## Results

**Fig. 1:**
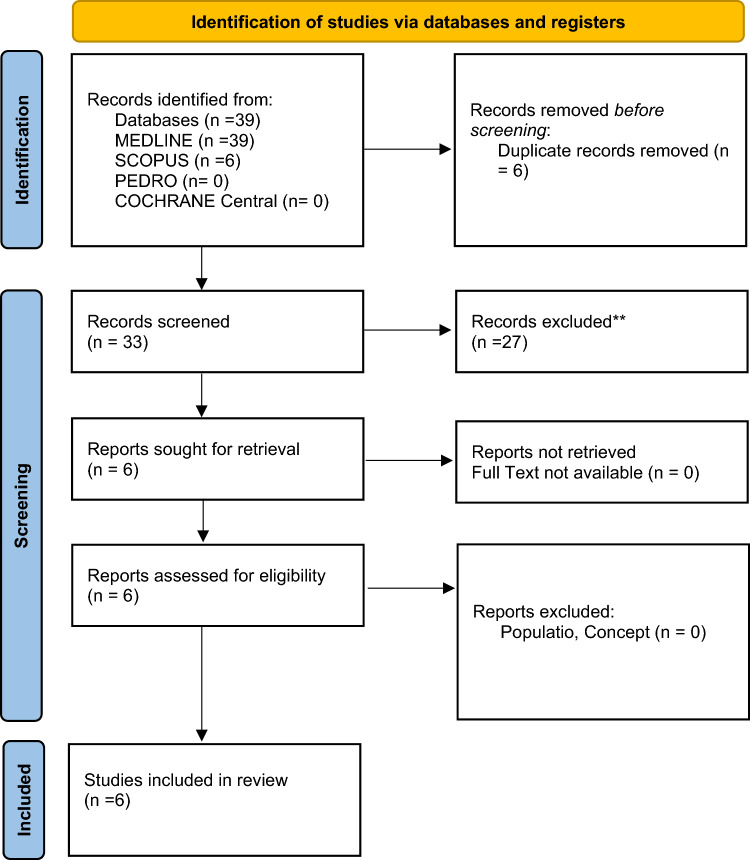
Preferred reporting items for systematic reviews and meta-analyses 2020 (PRISMA) flow diagram

As presented in the PRISMA 2020-flow diagram (Fig. 1), from 39 records identified by the initial literature searches, 33 were excluded and 6 articles were included.

In Marconi B et al., (2007), eight amputee subjects and nine healthy subjects were examined to investigate the functional reorganization in upper limb motor cortical maps following the loss of a leg. The amputees had undergone leg amputation above the knee several years prior, and all were using prostheses. The study aimed to understand the inhibitory relationship between foot and hand motor cortical representations and explore changes in cortical excitability during motor imagery tasks. The findings revealed that in healthy subjects, motor imagery of ankle movements resulted in a reduction of hand cortical map size and inhibition of hand muscle representations. However, in amputees, imagined ankle movements enhanced the excitability of the hand motor region instead of reducing its map size. Both amputees and healthy subjects exhibited a similar inhibition/facilitation pattern in forearm muscle cortical maps during motor imagery tasks. These results suggest that there is a breakdown in the inhibitory effects between foot and hand cortical representations following leg amputation. The study also highlighted the interlimb coordination mechanisms and the potential hardwired nature of isodirectional coupling between ipsilateral limbs. The findings contribute to our understanding of the neural mechanisms underlying interlimb coordination and provide insights for neurorehabilitation strategies. In summary, the study demonstrates that the loss of a leg induces functional reorganization in upper limb motor cortical maps and disrupts the inhibitory relationship between foot and hand representations. These findings have implications for rehabilitation approaches targeting interlimb coordination.

Coslett HB et al. (2010) study involved five groups of participants: bilateral leg pain (BLP), unilateral left leg pain (LLP), unilateral right leg pain (RLP), pain control (PC), and normal control (NC). Subjects with chronic leg pain and other types of pain were recruited, along with normal controls without chronic pain. The task required participants to indicate whether presented line drawings depicted a right or left foot. Stimuli included various angles of rotation. The results showed that subjects with leg pain were slower and less accurate compared to normal controls, and there was a group by rotation interaction, indicating that leg pain subjects were more affected by maximal rotation stimuli. Pain control subjects also showed slower and less accurate performance, but without a rotation by group interaction. There were rotation by group interactions between RLP and BLP groups and the PC group in the reaction time data. The findings suggested that the impaired performance of leg pain subjects was not solely due to the nonspecific effects of chronic pain. The foot laterality task was found to be a potentially useful adjunct for assessing leg pain. It was a brief task that did not require specialized equipment, and it provided an implicit measure of pain without explicit pain ratings. The task's implicit nature may minimize the influence of psychological factors on pain evaluation. Future evaluations should consider factors like depression, anxiety, and catastrophizing, and further explore the task's potential for assessing malingering or factitious pain disorders. The study also discussed the distinction between implicit and explicit movement representations and the relationship between pain and the sense of agency in action. The experience of chronic pain was suggested to alter the unconscious representation of the body involved in movement planning. The task's findings supported the hypothesis that the representation of imagined movements is mediated by a forward model that anticipates the sensory consequences, including pain. In summary, the foot laterality task showed differences in performance between subjects with leg pain and normal controls, providing a potential measure of pain associated with movement. The task's characteristics and implicit nature make it a promising tool for assessing leg pain in clinical settings.

King R et al. (2015) involved a 35-year-old female with Complex Regional Pain Syndrome (CRPS) who completed a Foot Laterality Recognition Task (LRT). Initially, she used a "3rd person" strategy, imagining another person's feet, and achieved good accuracy and comparable response times for left and right feet. However, when asked to imagine her own feet using a "1st person" strategy, her accuracy remained good but response times were significantly slower for right feet. The patient reported increased pain while using the "1st person" strategy. She then practiced LRTs at home and showed improvement in response times but maintained the same pattern of slower responses for right feet with the "1st person" strategy. Typically, LRTs are used to implicitly facilitate motor imagery, but this patient's alternative strategies challenged the therapeutic rationale. Biomechanical analysis confirmed that the patient's "1st person" strategy resulted in slower response times for awkward postures, especially for right feet, while her preferred "3rd person" strategy showed no such effects. These findings suggested distinct mechanisms for the two strategies. The case highlights that individuals can perform well on LRTs without engaging in motor imagery, which has implications for the use of LRTs in clinical settings. Practitioners should establish processes to carefully select images and analyze for the presence of biomechanical constraints to determine if patients are using a motor strategy or not. Current tools for LRTs may not provide sufficient control or analysis capabilities, and further research on alternative strategies in LRT performance is needed.

Shamsi F et al. (2020) study aimed to investigate whether motor imagery tasks can be distinguished in EEG data under pain-free and pain conditions. Four healthy, right-handed volunteers participated in the study. Painful stimuli were delivered to the dorsum of the left hand using a thermode, and temperatures associated with pain threshold and tolerance were determined for each subject. The main experiment consisted of 12 blocks, alternating between pain-free and under-pain conditions. During under-pain blocks, the thermode was heated to a random temperature within a specific range, while it was set to baseline temperature for pain-free blocks. Subjects performed motor imagery tasks based on visual cues indicating the type of task (right hand, left hand, foot, or tongue). EEG data were recorded using a 32-channel EEG system. The study utilized a functional connectivity-based method to extract functional connectivity graphs from EEG data and classify motor imagery tasks under pain-free and pain conditions. An LSTM artificial neural network was employed for classification. The results showed that the proposed method accurately differentiated pain-free motor imagery tasks from those performed under pain conditions. The classification accuracy was highest in the gamma frequency band. The findings suggest that the method can reliably determine whether motor imagery tasks are performed in pain-free or under pain conditions. Further research could explore the generalizability of the method to other tasks and investigate how the brain processes motor imagery tasks in the presence of pain. The results could contribute to the development of motor imagery-based brain–computer interfaces that adapt to pain conditions without affecting classification performance.

Shepherd M et al. (2020) case report describes the treatment of a 57-year-old female with Complex Regional Pain Syndrome (CRPS) following an ankle injury. The patient experienced progressive sensitivity, pain, and other symptoms in her right foot and ankle. She underwent a nine-month course of physical therapy (PT) that incorporated Graded Motor Imagery (GMI), Pain Neuroscience Education (PNE), and graded exposure. The patient's treatment progression included PNE to provide knowledge and understanding of pain, GMI to address the central nervous system and promote motor imagery, and graded exposure to gradually expose the patient to functional activities. Manual therapy was also integrated to facilitate her transition to weight-bearing and gait activities. The outcomes showed improvements in functional outcomes, pain levels, and psychosocial factors. The patient's functional milestone achievements were sustained at a 2-year follow-up, with no functional deficits related to her foot or ankle. The integration of PNE within GMI and graded exposure created an effective treatment approach, leading to significant improvements in the patient's condition. It is important to note that while GMI has shown promise in the treatment of CRPS, the evidence base is still limited, and more high-quality research is needed. The translation of GMI into clinical practice can also be challenging, requiring careful implementation and adaptation to individual patients. Overall, this case report highlights the potential benefits of combining PNE, GMI, and graded exposure in the treatment of CRPS, emphasizing the importance of addressing both physical and psychosocial factors in managing this complex condition.

Rio EK et al. (2021) study aimed to evaluate implicit motor imagery performance in individuals with Achilles tendinopathy (AT) compared to healthy controls. Participants with symptomatic mid-portion or insertional AT were recruited, and three different study sites implemented slightly different diagnostic criteria. The participants underwent a left–right judgment (LRJ) task using Recognise^©^, a program that presents pictures of hands and feet in various positions, and participants had to determine whether the image belonged to the left or right side of the body. The findings showed that there were no significant differences in LRJ performance between unilateral AT cases and healthy controls. The accuracy and reaction time for hand images were similar between the two groups. Regarding foot images, the primary analysis showed worse performance for unilateral AT cases compared to controls, suggesting impaired function of the working body schema for the foot. However, this impairment was not consistent across sensitivity analyses. The unaffected side of AT cases did not differ from the affected side. Exploratory analyses also found no differences between healthy controls and unilateral or bilateral AT cases. These findings suggest that motor imagery performance is not impaired in individuals with AT, at least in terms of LRJ tasks. However, the study had limitations, including not measuring other key outcomes such as proprioception, strength, sensorimotor processing, or cognitive ability. Further research is needed to explore the potential impairments in these areas and their relationship to motor imagery, movement, and disease progression in AT. The study also highlighted the importance of considering factors such as symptom duration, cognitive processes, and sensorimotor integration in understanding motor imagery performance in AT. It is unclear whether the size of implicit motor imagery impairment is relevant to movement dysfunction, pain levels, or response to treatment. The findings do not support the need to measure LRJ performance or target implicit motor imagery in the assessment or treatment of AT. The study's limitations include the combination of data from different study sites and the variability in data collection methods. Future studies should focus on standardizing protocols, including detailed demographic information, and investigating the role of factors such as activity level and chronicity in motor imagery performance in AT.

## Discussion

Motor imagery plays a crucial role in pain perception and rehabilitation, particularly in the context of foot pain. The reviewed articles provide valuable insights into the effects of motor imagery on foot pain and its underlying neural mechanisms. Marconi et al. (2007) explored the functional reorganization of upper limb motor cortical maps in amputees. They found that following leg amputation, there was a breakdown in the inhibitory relationship between foot and hand cortical representations. This suggests that the loss of a leg induces changes in motor cortical maps, potentially impacting interlimb coordination. These findings have implications for neurorehabilitation strategies targeting interlimb coordination in amputees. Coslett et al. [12] investigated the performance of individuals with leg pain in a foot laterality task. The results revealed that individuals with leg pain exhibited slower and less accurate performance compared to healthy controls. This task showed promise as an adjunct measure of pain associated with movement and may have clinical utility in assessing leg pain. The findings also shed light on the relationship between pain, implicit movement representations, and the sense of agency in action. King et al. [13] presented a case study of a patient with Complex Regional Pain Syndrome (CRPS). The patient exhibited distinct motor imagery strategies during a foot laterality task. The case highlighted the importance of analyzing biomechanical constraints and determining whether patients are using motor strategies or alternative approaches. This study emphasized the need for further research on alternative strategies in motor imagery tasks and their implications for clinical applications. Shamsi et al. [14] investigated the feasibility of distinguishing motor imagery tasks performed under pain-free and pain conditions using EEG data. The study employed a functional connectivity-based method and an artificial neural network for classification. The results demonstrated the potential of this approach to accurately differentiate between pain-free and pain-related motor imagery tasks. This research contributes to the development of motor imagery-based brain-computer interfaces that adapt to pain conditions. Shepherd et al. [15] presented a case report on the treatment of a patient with CRPS. The integration of Graded Motor Imagery (GMI), Pain Neuroscience Education (PNE), and graded exposure in the patient's physical therapy resulted in significant improvements in functional outcomes, pain levels, and psychosocial factors. This case report underscores the potential benefits of a multimodal approach in the management of CRPS and highlights the importance of addressing both physical and psychosocial factors in treatment. Collectively, these articles highlight the potential of motor imagery in foot pain management and neurorehabilitation. Motor imagery techniques can induce changes in cortical maps, facilitate interlimb coordination, and provide a measure of pain associated with movement. Furthermore, advances in EEG-based classification methods offer the potential for real-time assessment and adaptation of motor imagery tasks based on pain conditions. Integrating motor imagery approaches, such as GMI and PNE, into comprehensive treatment programs may enhance outcomes for individuals with foot pain conditions. While the reviewed articles provide valuable insights, further research is needed to explore the optimal protocols, individualized approaches, and long-term effects of motor imagery interventions in foot pain management. Future studies should also investigate the impact of factors such as psychological variables, cognitive processes, and sensorimotor integration on motor imagery performance and its clinical implications. Overall, motor imagery holds promise as a non-invasive, adjunctive approach for foot pain management, with potential applications in both clinical and rehabilitation settings.

This review emphasizes the potential of motor imagery in foot pain management. Motor imagery holds promise as a non-invasive approach in clinical and rehabilitation settings.

### Research implications and suggestions for clinical practice

The reviewed articles on motor imagery and foot pain have important implications for both research and clinical practice. These implications and suggestions are outlined below:

1. Further research on motor imagery: The findings from these studies highlight the need for additional research on motor imagery and its effects on foot pain. Future studies should explore the underlying neural mechanisms and the specific factors that influence motor imagery performance in individuals with foot pain. This research could include investigating the role of psychological variables, cognitive processes, sensorimotor integration, and the impact of chronicity and activity level on motor imagery abilities.
2. Standardization of protocols: To facilitate comparability and reproducibility across studies, it is crucial to establish standardized protocols for motor imagery tasks in the context of foot pain. Consistency in task design, instructions, and outcome measures will allow for more robust comparisons between studies and enhance the validity of findings.
3. Clinical assessment tools: The foot laterality task demonstrated in the studies shows promise as a potential clinical assessment tool for evaluating foot pain. Its implicit nature and simplicity make it a valuable adjunct measure for assessing pain associated with movement. Researchers should continue to refine and validate this task, considering factors such as depression, anxiety, catastrophizing, and its potential use in evaluating malingering or factitious pain disorders.
4. Integration into treatment approaches: The integration of motor imagery techniques, such as Graded Motor Imagery (GMI), into comprehensive treatment programs should be considered for individuals with foot pain conditions. Combining motor imagery with other interventions, such as Pain Neuroscience Education (PNE) and graded exposure, may yield better outcomes in terms of pain reduction, functional improvement, and psychosocial factors. However, further research is needed to establish the optimal protocols and individualized approaches for integrating motor imagery into treatment programs.
5. Neurorehabilitation strategies: The findings from studies on amputees and individuals with Complex Regional Pain Syndrome (CRPS) have implications for neurorehabilitation strategies. Understanding the functional reorganization in motor cortical maps and the breakdown of inhibitory effects between foot and hand representations can inform the development of targeted rehabilitation interventions. Strategies aimed at promoting interlimb coordination and restoring inhibitory mechanisms may enhance motor function and reduce pain-related disability in these populations.

In summary, the research on motor imagery and foot pain provides valuable insights into the neural mechanisms underlying pain perception and rehabilitation [26, 27]. The implications for research include further exploration of motor imagery, standardization of protocols, and the development of assessment tools. In clinical practice, integrating motor imagery techniques into treatment approaches and adopting neurorehabilitation strategies based on the findings can enhance outcomes for individuals with foot pain. Continued research in these areas will contribute to our understanding of motor imagery's role in pain management and improve the effectiveness of interventions targeting foot pain.

### Strengths and limitations

#### Strengths

1. Variety of study designs: The reviewed articles encompassed a range of study designs, including experimental studies, case reports, and clinical trials. This diversity enhances the comprehensiveness of the evidence and allows for a more holistic understanding of motor imagery and foot pain.
2. Sample diversity: The studies involved participants with various foot pain conditions, including amputees, individuals with Complex Regional Pain Syndrome (CRPS), and those experiencing chronic leg pain. This sample diversity enhances the generalizability of the findings to a broader population of individuals with foot pain.
3. Objective measures: Many of the studies utilized objective measures to assess motor imagery performance, such as reaction times, accuracy rates, and neurophysiological measures like EEG. These objective measures provide quantifiable data and enhance the reliability and validity of the findings.
4. Practical implications: The articles discussed potential implications for clinical practice, including the use of motor imagery tasks as assessment tools and the integration of motor imagery techniques into rehabilitation programs. These practical implications offer valuable insights for clinicians working with individuals experiencing foot pain.

#### Limitations

1. Small sample sizes: Some of the studies had relatively small sample sizes, which may limit the generalizability of the findings. Larger-scale studies with more participants would provide stronger evidence and increase the reliability of the results.
2. Heterogeneity in methodologies: There were variations in the methodologies across the studies, including differences in diagnostic criteria, task designs, and data collection methods. This heterogeneity makes it challenging to directly compare the findings and draw definitive conclusions.
3. Lack of standardized protocols: The absence of standardized protocols for motor imagery tasks and assessments in the context of foot pain limits the ability to make direct comparisons between studies. The development of standardized protocols would enhance the consistency and replicability of future research in this area.
4. Limited outcome measures: Some studies focused primarily on reaction times and accuracy rates during motor imagery tasks, neglecting other important outcome measures such as proprioception, strength, and functional outcomes. Incorporating a broader range of outcome measures would provide a more comprehensive assessment of motor imagery performance and its impact on foot pain.
5. Potential confounding variables: The reviewed articles primarily focused on motor imagery and foot pain, but other factors such as psychological variables, cognitive processes, and comorbidities may influence motor imagery performance and should be considered in future research.

Overall, while the reviewed articles provide valuable insights into the relationship between motor imagery and foot pain, it is important to acknowledge the limitations in sample sizes, methodologies, and outcome measures [28]. Addressing these limitations in future research will strengthen the evidence base and enhance our understanding of the topic.

### Answering evidence gap

The evidence presented in the reviewed articles highlights several gaps in our understanding of the relationship between motor imagery and foot pain. Addressing these gaps through further research will contribute to the advancement of knowledge in this area. Here are some potential avenues for future investigation:

1. Longitudinal studies: Conducting longitudinal studies would allow for the examination of motor imagery performance and its relationship to foot pain over time. This could help elucidate the causal direction of the relationship and provide insights into the dynamic nature of motor imagery in the context of pain.
2. Mechanistic studies: Further research is needed to explore the underlying mechanisms of motor imagery and its effects on foot pain. Neuroimaging techniques, such as functional magnetic resonance imaging (fMRI), could be employed to investigate the neural correlates of motor imagery in individuals with foot pain. Understanding the neurobiological mechanisms involved would enhance our knowledge of the processes underlying motor imagery and pain modulation.
3. Comparative studies: Comparative studies between different types of foot pain conditions, such as neuropathic pain, inflammatory pain, or musculoskeletal pain, would provide a more nuanced understanding of the specificities of motor imagery impairments in different pain populations. Comparing motor imagery performance across diverse foot pain conditions could also help identify commonalities or differences in underlying mechanisms.
4. Intervention studies: Investigating the efficacy of motor imagery interventions for managing foot pain is another important area for future research. Randomized controlled trials comparing motor imagery interventions to standard care or other active interventions could provide insights into the potential therapeutic benefits of incorporating motor imagery techniques in the treatment of foot pain.
5. Psychosocial factors: Given the influence of psychological and psychosocial factors on pain perception, future studies should consider exploring the impact of variables such as anxiety, depression, and pain catastrophizing on motor imagery performance in individuals with foot pain. Understanding the interplay between these factors and motor imagery could help inform personalized treatment approaches.
6. Standardized assessment protocols: Developing standardized protocols for assessing motor imagery performance in individuals with foot pain would facilitate comparison and replication of findings across studies. Consensus on measurement tools, task designs, and outcome measures would enhance the reliability and validity of future research in this area.

In summary, there are several gaps in the current evidence regarding motor imagery and foot pain. Future research should focus on longitudinal studies, mechanistic investigations, comparative analyses, intervention trials, examination of psychosocial factors, and the establishment of standardized assessment protocols [22]. By addressing these evidence gaps, we can deepen our understanding of the role of motor imagery in foot pain and inform the development of effective interventions for individuals experiencing foot pain.

### Clinical practice

The findings from the reviewed articles have implications for clinical practice in the assessment and management of foot pain. Here are some key points to consider:

1. *Assessment tools*: The use of motor imagery tasks, such as the foot laterality recognition task, can provide valuable insights into the implicit motor imagery abilities of individuals with foot pain. Integrating these tasks into the assessment process can help clinicians gain a better understanding of motor imagery impairments and their potential role in the pain experience.
2. *Individualized treatment plans*: The evidence suggests that motor imagery impairments can vary among individuals with foot pain. Therefore, treatment plans should be tailored to each patient's specific needs and abilities. Assessing motor imagery performance and considering factors like pain severity, duration, and psychosocial factors can aid in the development of individualized treatment approaches.
3. *Motor imagery interventions*: The use of motor imagery interventions may hold promise for managing foot pain. Implementing techniques such as graded motor imagery, which involves progressively engaging in imagined movements, can potentially modulate pain perception and improve functional outcomes. Integrating motor imagery interventions alongside other evidence-based treatments, such as physical therapy and pain education, may optimize outcomes for individuals with foot pain.
4. *Patient education*: Educating patients about the potential benefits of motor imagery and its role in pain modulation can empower them to actively participate in their own care. Providing patients with a clear understanding of the underlying mechanisms and rationale behind motor imagery interventions can enhance treatment adherence and engagement.
5. *Interdisciplinary collaboration*: Foot pain often requires a multidisciplinary approach. Collaborating with healthcare professionals from various disciplines, such as physiotherapists, psychologists, and pain specialists, can facilitate comprehensive assessment and management of foot pain. Incorporating motor imagery techniques into interdisciplinary treatment plans can address the physical, psychological, and cognitive aspects of foot pain.
6. *Monitoring progress*: Regular assessment of motor imagery performance and its impact on pain levels and functional outcomes is crucial. Tracking changes in motor imagery abilities over time can help evaluate treatment efficacy and make necessary adjustments to the intervention plan. Objective measures, such as reaction times and accuracy on motor imagery tasks, can provide valuable data for monitoring progress.

It is important to note that while the evidence suggests the potential benefits of motor imagery in foot pain management, further research is needed to establish optimal protocols, explore long-term effects, and determine the specific patient populations that may benefit most from motor imagery interventions.

## Conclusions

In conclusion, the reviewed articles provide evidence on the role of motor imagery in foot pain. The findings suggest that individuals with foot pain may experience impairments in motor imagery abilities, such as reduced accuracy and slower response times. These impairments may be related to functional reorganization in the motor cortical maps following leg amputation or the presence of chronic pain. However, the results are not consistent across all studies, highlighting the need for further research in this area. The implications for clinical practice include the use of motor imagery tasks as assessment tools, the development of individualized treatment plans targeting motor imagery impairments, and the integration of motor imagery interventions alongside other evidence-based treatments. Patient education, interdisciplinary collaboration, and regular monitoring of progress are also important considerations. Overall, while motor imagery shows promise as a tool for understanding and managing foot pain, more research is needed to establish optimal protocols, explore long-term effects, and determine the specific patient populations that may benefit most from motor imagery interventions.

## Data Availability

Data and materials related to this study are available upon polite request.
